# Rapid Identification of Alien Chromosome Fragments and Tracing of Bioactive Compound Genes in Intergeneric Hybrid Offspring Between *Brassica napus* and *Isatis indigotica* Based on AMAC Method

**DOI:** 10.3390/ijms26052091

**Published:** 2025-02-27

**Authors:** Yanhong Guo, Yutian Han, Jinxiang Gao, Xianhong Ge, Yanqing Luo, Kaiqin Zhao, Genze Li, Feng Zu, Xiaomao Cheng

**Affiliations:** 1 School of Agriculture, Yunnan University, Kunming 650504, China; 13278725781@163.com; 2 Yunnan Key Laboratory of Genetic Improvement of Herbal Oil Crops, Industrial Crops Research Institute, Yunnan Academy of Agricultural Sciences, Kunming 650225, China; hanyutian1998@163.com (Y.H.); gaojx@yaas.org.cn (J.G.); luoyq@yaas.org.cn (Y.L.); zkq@yaas.org.cn (K.Z.); lgz@yaas.org.cn (G.L.); 3 Engineering Technology Research Center of National Forestry and Grassland Administration on Southwest Landscape Architecture, College of Landscape Architecture and Horticulture Sciences, Southwest Forestry University, Kunming 650224, China; 4 College of Plant Science and Technology, Huazhong Agricultural University, Wuhan 430070, China; gexianhong@mail.hzau.edu.cn

**Keywords:** *Brassica napus*, *Isatis indigotica*, distant hybridization, intron polymorphism (IP), SSR, AMAC method, alien chromosome

## Abstract

Distant hybridization between *Brassica napus* and related genera serves as an effective approach for rapeseed germplasm innovation. *Isatis indigotica*, a wild relative of *Brassica*, has emerged as a valuable genetic resource for rapeseed improvement due to its medicinal properties. This study employed anchor mapping of alien chromosomal fragment localization (AMAC) method to efficiently identify alien chromosomal fragments in the progeny derived from distant hybridization between *I. indigotica* and *Brassica napus*, ‘Songyou No. 1’. Based on the AMAC method, we developed 193,101 IP and SSR markers utilizing the *I. indigotica* reference genome (Woad-v1.0). Through Electronic-PCR analysis against the *Brassica* and *I. indigotica* pan-genome, 27,820 specific single-locus (SSL) IP and SSR markers were obtained. Subsequently, 205 pairs of IP primers and 50 pairs of SSR primers were synthesized randomly, among which 148 pairs of IP markers (72.20%) and 45 pairs of SSR markers (90%) were verified as SSL molecular markers for the *I. indigotica* genome with no amplification product in four *Brassica* crops. These 193 SSL markers enable precise identification of one complete I6 chromosome and three chromosomal fragments (I1:1.17 Mb, I5:2.61 Mb, I7:1.11 Mb) in ‘Songyou No. 1’. Furthermore, we traced 32 genes involved in bioactive compound biosynthesis within/near these alien segments in ‘Songyou No. 1’ and developed seven functional markers. This study not only validates the efficacy of SSL markers for detecting exogenous chromatin in intergeneric hybrids but also provides valuable insights for the precise identification and mapping of desired chromosomal fragments or genes embedded in the derivatives from distant hybridization and potential applications in marker-assisted breeding for medicinal plant via distant hybridization strategy between *I. indigotica* and *Brassica* crops.

## 1. Introduction

Crop improvement for increased yield, resistance against abiotic/biotic stresses, and other desired traits over time is imperative. The combination of sought-after traits from different species’ parents to one offspring is evidently the most common improvement method and is known as distant hybridization. Distant hybridization between *Brassica* and related genera serves as an effective approach for germplasm innovation in rapeseed breeding, which also plays a significant role in the natural evolution of *Brassica* species [1]. *Isatis indigotica* Fort (2n = 14, II) belongs to the Brassicaceae family, commonly known as “Banlangen” for its dried roots [2]. It is a traditional Chinese herbal medicine with properties for clearing heat, detoxifying, preventing colds, and soothing the throat [3]. Pharmacological studies have demonstrated *I. indigotica*’s therapeutic potential, including antibacterial, anticancer, antiviral, antipyretic, anti-inflammatory, choleretic, anti-endotoxin, and immune-enhancing activities [4,5]. Besides pharmacological benefits, *I. indigotica* also exhibits resistance against tobacco mosaic virus (TMV) [6] and the fungal pathogen *Sclerotinia sclerotiorum*, which causes stem rot in rapeseed [2]. The medicinal properties primarily derive from three bioactive compound classes: terpenoids, lignans, and indole alkaloids, and a total of 59, 66, and 32 genes involved in the biosynthetic pathways of the above three active compounds have been respectively identified and annotated in the reference genome of *Isatis indigotica*, ‘Woad-v1.0’ [4].

Currently, *I. indigotica* has been extensively applied for the genetic improvement of *Brassica* crops through distant hybridization. Du et al. [7] successfully generated somatic hybrids between *B. napus* and *I. indigotica* Fort. via somatic cell fusion. Subsequently, a complete set of *B. napus–I. indigotica* monosomic alien addition lines (MAALs) were created [8,9]. Notably, three disomic alien additions (Dd, Df, and Dg) derived from crosses between *B. napus*, ‘Huashuang No. 3’ (2n = 38, AACC) and *I. indigotiga* (2n = 14, II) exhibit resistance to highly pathogenic H5N6 avian influenza virus, with efficacy comparable to the clinical antiviral drug Oseltamivir (Tamiflu^®^) [10]. The Dd addition line, certified as ‘Lancai No. 1.’ (Variety Certification No. Jingpinjian Cai 2014032) by the Beijing Seed Management Station, displayed in vitro inhibitory activity against the Influenza A virus strain A/PR/8/34 (H1N1). This discovery pioneered the development of antiviral *Brassica* vegetables, including *B. oleracea* and canola derivatives [11]. The MAAL Mg line served as the male parent in developing the antiviral addition line ‘Songyou No. 1’ through crossed with ‘Huashuang No. 3’ (*B. napus*, female parent) [11]. Subsequent hybridization between ‘Songyou No. 1’ and ‘Chuan A-3’ (*B. napus*) yielded the improved ‘Songyou No. 2’ [12]. This innovation in *B. napus* germplasm resources demonstrates multifunctional potential for utilization in vegetable production, livestock feed formulations, and pharmaceutical industries [10,12,13].

With the ongoing development of a superior intergeneric hybrid between *I*. *indigotica* and *Brassica* crops, how to rapidly and efficiently identify and map the introgressed *I. indigotica* chromosomal fragments has become increasingly crucial. Current identification techniques for alien chromatin in distant hybridization progenies include: (1) phenotypic trait evaluation, (2) cytogenetic analysis (karyotyping, banding patterns, fluorescence/genomic in situ hybridization), and (3) molecular marker systems. Among these, molecular markers serve as indispensable tools due to their environmental independence, high throughput, reproducibility, and operational efficiency [14,15]. Previous studies have demonstrated the use of *I*. *indigotica* genome-specific molecular markers for the identification of its distant hybridization progenies with *B. napus* [8]. However, only a few markers between specific species of *I*. *indigotica* and *B. napus* have been developed, which do not yet meet the universal requirements for identifying *I*. *indigotica* exogenous fragments in intergeneric *Brassica* hybrids. Therefore, developing comprehensive, genome-specific molecular markers with robust cross-hybrid validation will advance germplasm innovation and utilization in *I*. *indigotica–Brassica* distant hybridization.

Over the past decade, a high-quality reference genome for diploid progenitors and allopolyploid species within the *Brassica* genus has been systematically characterized and driven by high-throughput sequencing technology [16,17,18,19]. These resources have enabled pan-genome analyses that delineate species-level structural variations and the presence/absence of gene polymorphisms across *Brassica* germplasm [20]. As a collective representation of all genomic sequences within a species or genus, pan-genomes provide critical insights for identifying introgressed chromatin in distant hybrids. Traditional single-reference approaches often fail to detect species-specific genetic variation in hybrid progeny, whereas pan-genome comparisons enable comprehensive identification of donor-derived sequences through muti-reference analysis [20]. In recent years, genetic variation datasets have been established for *Brassica* crops such as *B. napus*, *B. oleracea*, and *B. rapa*. Based on pan-genome information, significant agronomic traits and candidate genes related to species domestication have been identified [20,21,22,23]. Building on this foundation, we previously proposed an anchor mapping of alien chromosome fragment (AMAC) method—a five-stage pipeline for rapid detection and localization of introgressed chromosomal segments: (1). Donor genomic marker development: Design whole-genome molecular markers (IP and/or SSR) targeting the distant hybridization donor species utilizing the newest reference genome sequence. (2). SSL markers prediction: Perform in silico PCR screening against recipient species’ pan-genomes to predict species-specific single-locus (SSL) markers. (3). Experimental validation: Verify marker specificity through PCR amplification and agarose gel electrophoresis across various parental lines. (4). Hybrid screening: Deploy validated SSL markers to detect alien chromatin in individual offspring plants derived from distant hybridization. (5). Boundary refinement: Iteratively screen and validate new SSL markers, flanking the previously validated SSL markers to precisely define the size of the introgressed fragments [24]. This method has successfully been applied to identify the radish (Raphanus sativus) chromosome segment and locate the *ORF* gene in the progeny of rape-radish intergeneric hybrid [24].

In this study, we implemented our established AMAC methodology to develop and partially verify whole-genome SSL molecular markers for *I. tinctoria* through systematic screening against multiple reference genomes: the *B. napus* pan-genome [20], *B. oleracea* [21], *B. rapa* [23], *B. juncea* [22], and *I. indigotica* [4]. Then, these genome-wide SSL markers were used to detect the source and sizes of I. tinctoria chromosomal fragments and trace the gene clusters associated with medicinally valuable compounds in ‘Songyou No. 1’. The results provide efficient and reliable molecular markers for the identification of *I. tinctoria*-*Brassica* intergeneric hybrid offspring and facilitate targeted introgression breeding in *B. napus* germplasm improvement.

## 2. Results

### 2.1. Development of I. indigotica Whole-Genome Intron Polymorphism (IP) and SSR Markers

**Figure 1:**
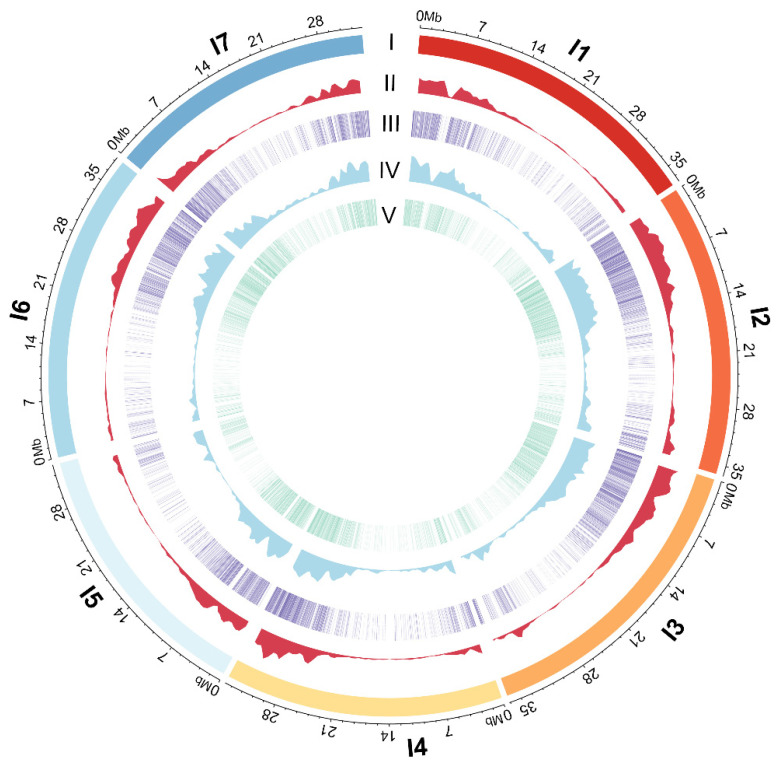
Circle diagrams illustrating the distribution and density of IP and SSR primers developed based on the Woad-v1.0 genome. The outermost circle denotes the physical size (Mb) of Woad-v1.0, I1–I7 means chromosome name, and each is indicated by different colors. Circles II and III show the distribution positions and densities of designed SL-IP and SL-SSR Makers, respectively. Circles IV and V show the distribution density and location of developed SSL-IP and SSL-SSR Makers.

A total of 30,323 genes were annotated in the genome file of *I. indigotica* (Woad-v1.0) [4]; 29,784 of these contained 132,473 introns in total, with an average of 4.45 introns per gene. 16,311, 20,012, 20,907, 16,852, 14,361, 17,868, and 17,258 introns were detected on each chromosome, I1-I7. Moreover, 8904 introns were found on the Contigs (Figure 1, Table S1).

**Figure 2:**
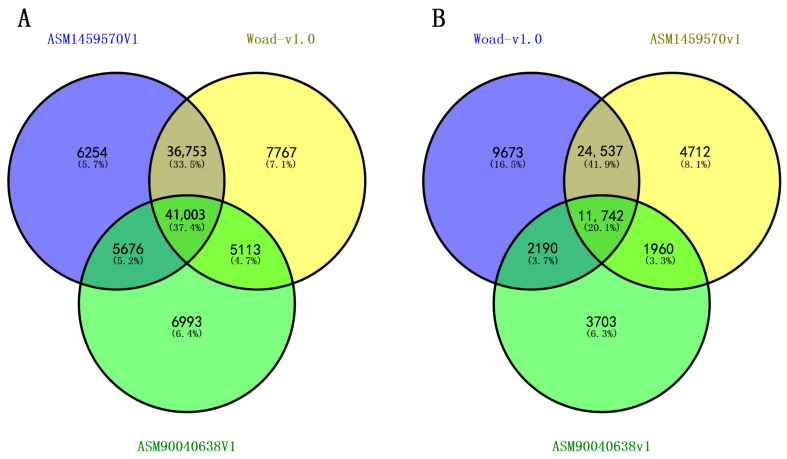
The Venn diagram representing IP (**A**) and SSR (**B**) primer statistics for single locus amplification via e-PCR analysis across three *I. indigotica* reference genomes.

A total of 128,302 (96.85%) introns with a length of 500 bp or less were used for the development of genome-wide IP markers, resulting in the successful development of IP primers for 120,302 intron loci, accounting for 90.81% of all introns. Then, 90,636 pairs of IP primers anticipatedly amplified only one locus in the Woad-v1.0 genome with method of electronic PCR (ePCR) in silico, which also were subjected to ePCR analysis in the *I. indigotica* reference genomes ASM1459570v1, and ASM90040638v1, resulting in expected PCR amplification products for 89,686, and 58,785 pairs of IP primers (Table 1), respectively. In the *I. indigotica* genome ASM1459570v1, there are 89,686 marker pairs expected to amplify one locus, 14,837 marker pairs expected to amplify two loci, 3556 markers expected to amplify three loci, and 3357 marker pairs expected to amplify more than three loci. In the *I. indigotica* genome ASM90040638v1, there are 58,785 markers expected to amplify one locus, 12,364 markers expected to amplify two loci, 2359 markers expected to amplify three loci, and 1229 marker pairs expected to amplify more than three loci (Table 1). A total of 41,003 IP primer pairs were identified that were expected to amplify a single locus across all three *I. indigotica* reference genomes (Figure 1 and Figure 2A, Table S2). These IP primers are predicted to be genome-wide SL-IP markers specific to *I. indigotica*.

A total of 83,589 SSR loci were identified in *I. indigotica* reference genome Woad-v1.0, of which 72,764 loci were successfully developed with primers. Among these, 48,142, 42,951, and 19,595 SSR primer pairs were expected to amplify a single locus in the three respective *I. indigotica* reference genomes (Table 1). The results of ePCR showed that a total of 11,742 pairs of SSR primers were expected to amplify a single locus across all reference genomes, including Woad-v1.0, ASM1459570v1, and ASM90040638v1 (Figure 1 and Figure 2B, Table S3). These 11,742 pairs of primers are expected to serve as SL-SSR markers for the *I. indigotica* genome.

### 2.2. e-PCR Analysis for Whole-Genome SSL Markers in I. indigotica as Compared to the Pan-Genome of Brassica Species

**Figure 3:**
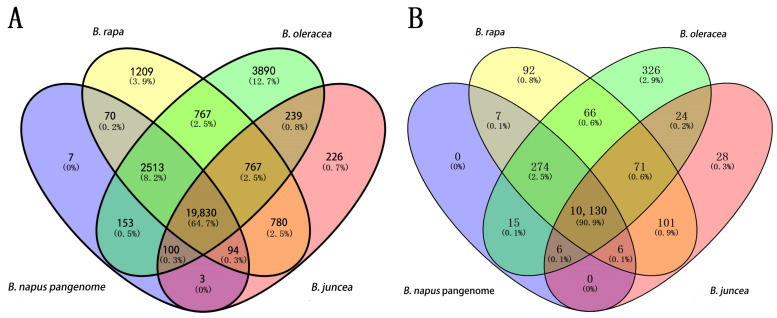
Venn diagram representing numbers of e-PCR product-free IP (**A**) and SSR (**B**) markers in four *Brassica* genomes.

A total of 41,003 *I. indigotica* whole-genome SL-IP markers obtained from previous analysis were further subjected to e-PCR analysis across the genomes of several *Brassica* species, including *B. napus*, *B. rapa*, *B. oleracea* and *B. juncea*. The results revealed that 18,233 *I. indigotica* whole-genome SL-IP markers were predicted to amplify in the *B. napus* pan-genome, 14,973 pairs in the *B. rapa* genome, 12,744 pairs in the *B. oleracea* genome, and 18,964 pairs in the *B. juncea* genome (Table 2). In contrast, 22,770, 26,030, 28,259, and 22,039 pairs of *I. indigotica* whole-genome SL-IP markers were predicted to have no amplification products in the above four *Brassica* species genomes, respectively. By taking the union of the reverse selection results, it was evident that 19,830 pairs of *I. indigotica* whole-genome SL-IP markers were predicted to have no PCR amplification products in any of four *Brassica* species (Figure 1 and Figure 3A, Table S4). These 19,830 pairs of IP markers are designated to be whole-genome SSL-IP markers unique to *I. indigotica*.

Similarly, 11,742 pairs of *I. indigotica* whole-genome SL- SSR markers obtained from the previous analysis were analyzed with e-PCR across the genomes of 4 *Brassica* species, including *B. napus*, *B. rapa*, *B. oleracea*, and *B. juncea*. The results showed that 1376 pairs of *I. indigotica* SL-SSR markers were predicted to amplify in the *B. juncea* reference genome, 1304 pairs in the *B. napus* pan-genome, 995 pairs in the *B. rapa* genome, and 830 pairs in the *B. oleracea* genome (Table 2). By comparison, 10,366, 10,438, 10,747, and 10,912 pairs of *I. indigotica* whole-genome SL-SSR markers were predicted to have no amplification products in the above four *Brassica* species, respectively. By taking the union of the reverse selection results, it was clear that 10,130 pairs of *I. indigotica* whole-genome SL-SSR markers were predicted to have no PCR amplification products in all four *Brassica* crops (Figure 1 and Figure 3B, Table S5). These 10,130 pairs of SSR markers were designated as specific whole-genome SSL-SSR markers for *I. indigotica*.

### 2.3. PCR Validation and Map Construction for I. indigotica Whole-Genome SSL-IP and SSL-SSR Markers

**Figure 4:**
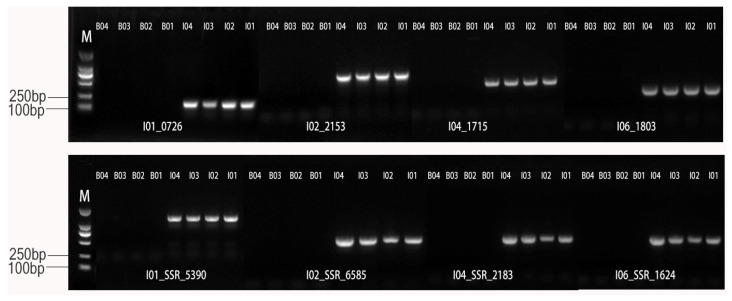
Screening of *I. indigotica* genome SSL markers against *Brassica* species. Note: M stands for DL2000 DNA Marker; B01–B04 represents four *Brassica crops*; I01–I04 represents four *I. indigotica* genomes.

**Figure 5:**
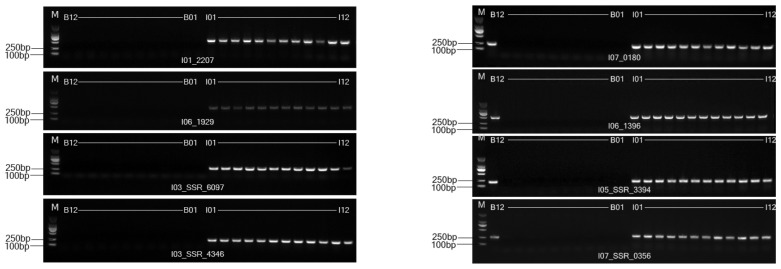
PCR validation for *I. indigotica* genome SSL markers against *Brassica* and *I. indigotica* genotypes. M stands for the marker; B01-B12 represents 12 *Brassica crops*, and B12 represents *B. napus* addition line ‘Songyou No. 1’; I01-I12 represents 12 *I. indigotica* materials.

**Figure 6:**
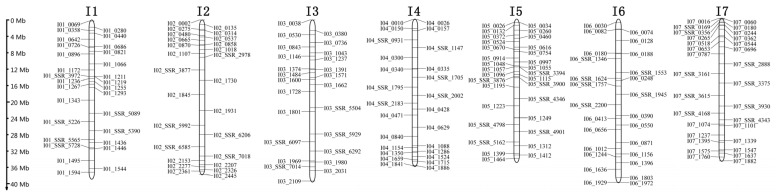
193 genome SSL-SSR and SSL-IP markers map of *I. indigotica*, the unit is Mb.

In order to validate the predicted *I. indigotica* genome SSL-IP and SSL-SSR markers, primers were selected according to the expected results of the amplifying site and size from the e-PCR analysis. A total of 205 pairs of IP primers and 50 pairs of SSR primers were synthesized. Initial screening for specificity was conducted using these primers across four *I. indigotica* genomes and four *Brassica* genomes (Figure 4). Differential markers identified were further validated in a small-scale test involving 12 *I. indigotica* samples and 12 *Brassica* crop samples (Figure 5). The results showed that some markers amplified in the 12 *I. indigotica* samples but did not amplify in any of the 12 *Brassica* crop samples (Figure 5). Through the validation process, a total of 193 genomes SSL-IP and SSL-SSR markers (Table S6) were screened for *I. indigotica*, including 148 IP markers and 45 SSR markers. These markers were distributed across the *I. indigotica* genome with an average density of 0.64 markers per Mb (Table 3). The relatively even distribution of these 193 SSL-IP and SSL-SSR markers across the seven chromosomes of *I. indigotica* facilitated the development of a comprehensive whole-genome SSL marker map for the species (Figure 6).

### 2.4. Tracing of Bioactive Compounds Genes in B. napus Addition Line ‘Songyou No. 1’ and Development of Functional Markers

**Figure 7:**
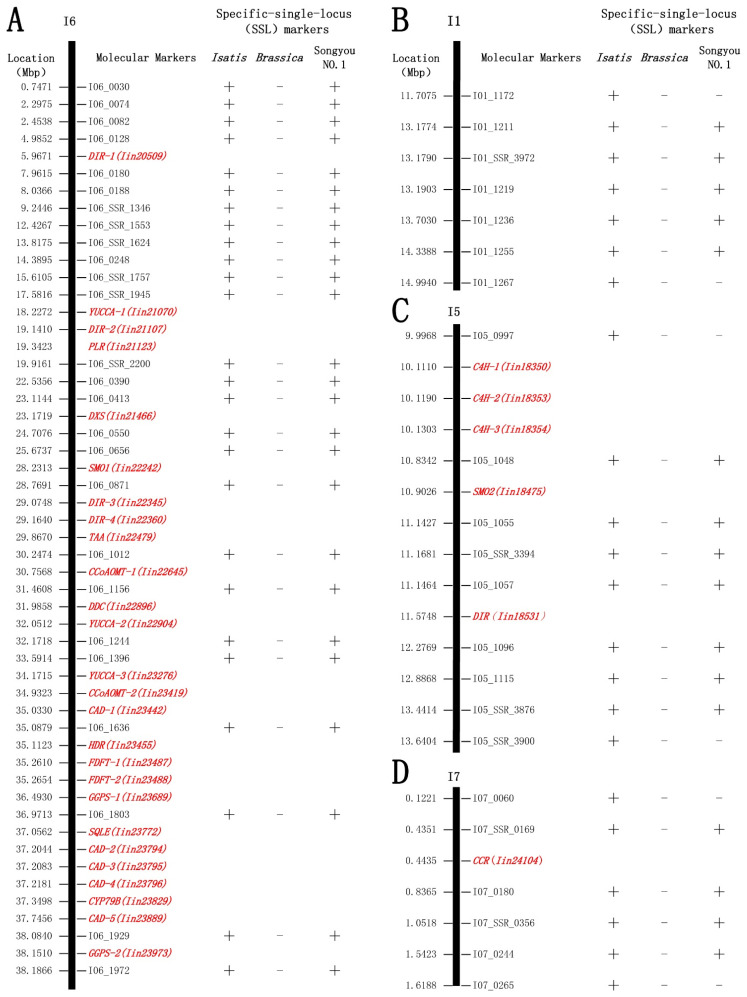
**SSL** Marker maps of *I. indigotica* alien chromosome and fragments from *I. indigotica* genome in ‘Songyou No. 1’. (**A**–**D**) represent one complete chromosome (I6), and three chromosomal fragments (I1:1.17 Mb, I5:2.61 Mb, I7:1.11 Mb) *; Isatis* represents 12 *I. indigotica* materials; *Brassica* represents 11 *Brassica materials*; “+” represents an *I. indigotica* fragment; ”-” represents absence of *I. indigotica* fragment; the red font represents genes related to medicinal values.

Unique markers for the *I. indigotica* genome were used to detect alien chromosomal fragments in *B. napus* addition line ‘Songyou No. 1’. The results showed that a complete *I. indigotica* chromosome I6 (g) was present in the hybrid progeny of ‘Songyou No. 1’, which is consistent with previous GISH analysis findings in *I. indigotica* hybrid progeny [8] (Figure 7A). Additionally, three *I. indigotica* chromosome segments I1 (f), I5 (c), and I7 (a) (Table S1) were identified with sizes of 1.17 Mb (Figure 7B), 2.61 Mb (Figure 7C), and 1.11 Mb (Figure 7D).

To analyze whether the alien fragments of *I. indigotica* in ‘Songyou No. 1’ contain genes responsible for the biosynthesis of bioactive compounds, we examined its biosynthetic pathways according to the reference genome annotation [4]. Thirty-two relevant genes are distributed across chromosomes I5 (c), I6 (g), and I7 (a) (Table S1), with the highest number (26 genes) found on chromosome I6 (g). These genes were primarily categorized into three groups: Terpenoids and sterols biosynthetic pathways, Lignans and flavonoids biosynthetic pathways, and Indole alkaloids biosynthetic pathways. Specifically, the terpenoids and sterols biosynthetic pathways involved 9 genes, the lignans and flavonoids biosynthetic pathways included 17 genes, and the indole alkaloids biosynthetic pathways contained 6 genes (Figure 7, Table 4). The identification of genes related to the biosynthesis of bioactive compounds on the aforementioned alien chromosomal fragments suggests that ‘Songyou No. 1’ has potential antiviral activity. Furthermore, on chromosome I5 (c), the SSL-IP marker I05_1048 was located only about 68 Kb away from the SM02 (Iin18475) gene, while I05_1057 is situated about 428 Kb from the DIR (Iin18531) gene. These markers can serve as tightly linked loci for these two genes and may be utilized in molecular marker-assisted breeding in the future.

**Figure 8:**
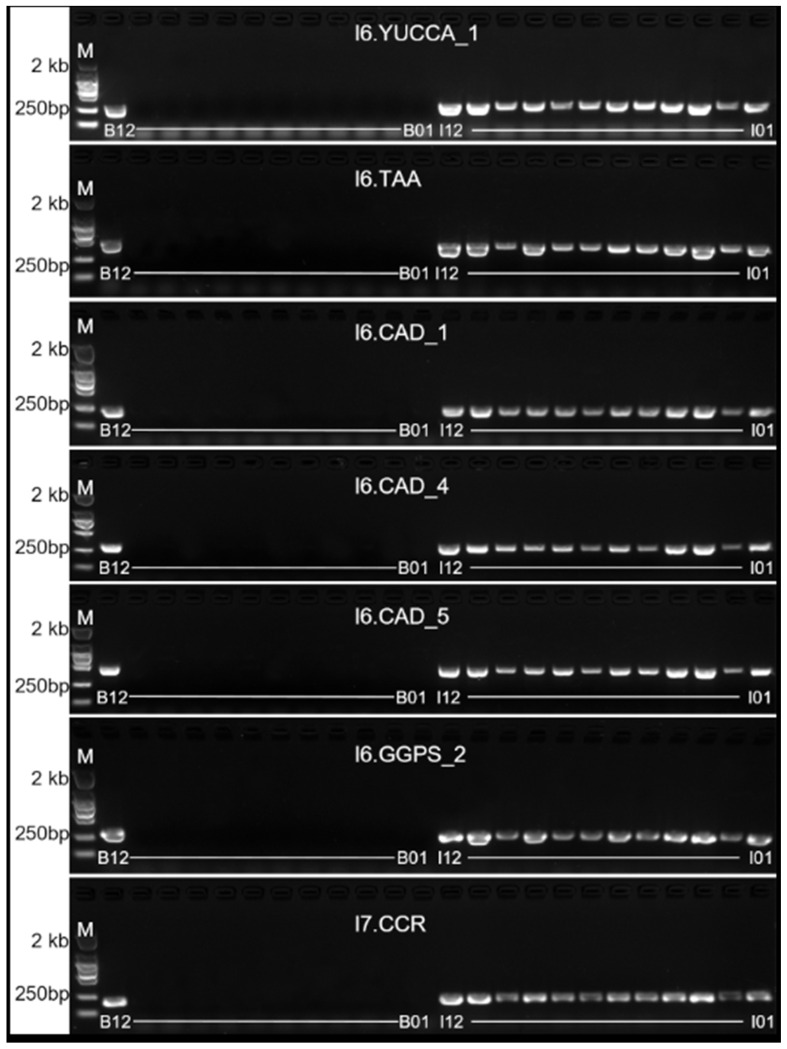
PCR validation of functional markers for bioactive genes in the alien chromosome fragments from *I. indigotica* genome in ‘Songyou No. 1’. M stands for marker; B01-B11 represents 11 *Brassica crops*; B12 represents ‘Songyou No. 1’; I01-I12 represents 12 *I. indigotica* materials.

Based on the identification of 32 genes associated with the production of bioactive substances, genome SSL-IP markers were developed. Among these, SSL-IP markers were successfully developed for 7 genes, with 6 markers located on chromosome I6 and 1 marker on chromosome I7 (Table 5, Figure 8). These genome SSL-IP markers can be utilized as functional markers in the future to detect these genes.

## 3. Discussion

Molecular marker technology has been widely adopted in various aspects of crop breeding, including variety identification, genetic map construction, gene mapping and cloning [25], germplasm improvement [15]^,^ and marker-assisted selection [26]. In *I. indigotica*, molecular marker technology played a significant role in identifying distant hybrid progeny, exploring genetic diversity, and identifying germplasm [8,27,28]. However, there are few reports on the development of specific molecular markers for the *I. indigotica* genome and systematic approaches. The advancement of whole-genome sequencing, along with the availability of robust in silico tools, can accelerate the development of low-cost, highly efficient gene-associated functional molecular markers for gene mapping, crop breeding, and germplasm resource identification. Introns, once considered non-coding DNA, are now recognized for their critical roles in regulating gene expression [29]. By leveraging publicly available genome sequences, this study identified introns across the entire genome and explored their polymorphisms as molecular markers in plants. Among simple PCR-based markers, IPs are gene-specific, often hypervariable, environmentally neutral, and co-dominant, with high transferability across related species [30]. Genome-wide intron-derived polymorphic markers have been reported in rice [31], foxtail millet [32], sorghum [33], chickpea [34], *Macrotyloma* spp. [35] and tea plant [36] were reported. The current study deployed self-developed IP3.0 software to develop *I. indigotica* whole genome SL-IP and SL-SSR markers against the four different published genomes of *I. indigotica.* These *I. indigotica* genome-identified markers were then subjected to the e-PCR analysis against the *Brassica* genomes. The markers that failed to give any amplifications against the *Brassica* genomes and only resulted in amplifications for a single locus against the *I. indigotica* genome were designated as the genome SSL markers for *I. indigotica* genomes.

This study successfully constructed a high-resolution SSL marker map for *I. indigotica* by integrating genome-wide SSL-IP and SSL-SSR markers, effectively resolving longstanding challenges associated with marker distribution bias and coverage gaps in conventional systems [37]. The strategic use of anchor markers further enabled rapid and precise identification of alien chromosomal insertion fragments from *I. indigotica* in hybrid genomes, achieving unprecedented specificity in detecting heterologous chromosomal segments. This advancement significantly improves the efficiency of tracking exogenous DNA in complex hybrid backgrounds, as exemplified by the successful characterization of *I. indigotica* chromosomal fragments in the *B. napus* hybrid ‘Songyou No. 1’ [3]. Notably, this approach addresses a critical gap in plant genomics: while most prior studies relied on limited differential markers derived from pairwise species comparisons [38], our pan-genome-informed strategy leverages comprehensive genomic data from the *Brassica* genus and *I. indigotica* to develop universally applicable markers. These markers fulfill the pressing need for robust tools to identify *I. indigotica*-derived fragments across diverse intergeneric hybrids within the *Brassica* lineage [38]. It meets the general requirements for identifying exogenous fragments of *I. indigotica* in a wide range of intergeneric hybrids between *I. indigotica* and the *Brassica* genus. At the same time, a relatively systematic identification method for molecular markers of exogenous chromosomal fragments in *I. indigotica* has been established, which will provide important references for the identification of heterologous chromosomes in the progeny of distantly hybridized crops involving *I. indigotica*.

Distant hybrids are important intermediate materials for creating alien addition lines, and the hybrids produced through distant hybridization may experience severe segregation. Therefore, how to quickly and accurately identify the exogenous chromosomal fragments in the genomes of the offspring of elite distant hybrid lines is crucial for subsequent genetic and application research. While conventional cytogenetic techniques such as genome in situ hybridization (GISH) and fluorescence in situ hybridization (FISH) have been widely used to detect chromosomal variations [39], their utility is constrained by three key limitations: technical complexity, prolonged experimental timelines, and inability to resolve the precise size or boundaries of introgressed fragments [40,41]. These constraints render them impractical for high-throughput screening of large hybrid populations—a critical requirement in modern breeding programs. In contrast, molecular markers are widely used in the creation and identification of distant hybrid alien addition lines in the *Brassicaceae* family due to their stability, high sensitivity, large number, reliable repeatability, and insensitivity to spatiotemporal environmental factors. For instance, a complete set of the *Brassica napus*-*Raphanus sativus* disomic addition lines was successfully identified using RAPD markers [42]. Compared to RFLP, RAPD, and AFLP markers, SSR markers exhibit richer polymorphism and simpler operation, making them favored by researchers. Li et al. [43] identified the sixth disomic addition line of the cabbage-*Brassica* napus C genome using C genome-specific SSR markers, Zhu et al. [44] identified a complete set of AA+1C1-9 MAALs using C subgenome-specific SSR markers, and Tan et al. identified seven *Brassica oleracea*-*nigra* MAALs using black mustard chromosome-specific SSR markers [45]. Building on this foundation, Kang et al. [8] employed chromosome-specific SSR markers to establish a complete set of *B. napus–Isatis indigotica* MAALs, validated through GISH. While these studies underscore the utility of SSR markers, their reliance on low-density marker systems restricts resolution and fails to address genome-wide coverage gaps. Our study advances the field through integrated SSL-IP and SSL-SSR markers, achieving unprecedented precision in tracking alien chromosomal fragments. Unlike prior approaches limited to pairwise species comparisons [38], our pan-genome-informed strategy utilizes genus-wide *Brassica* genomic data to develop markers with cross-species applicability, ensuring robust identification of *I. indigotica*-derived segments across diverse intergeneric hybrids. The hybrid line ‘Songyou No. 1’ (*B. napus–I. indigotica*) exemplifies this approach’ efficacy: SSL-IP and SSL-SSR markers not only confirm intact *I. indigotica* chromosomes (as previously shown by GISH [8]) but also precisely mapped three discrete chromosomal fragments—I1 (1.17 Mb), I5 (2.61 Mb), and I7 (1.11 Mb)—within the hybrid genome. This dual-marker system enhances resolution through increased marker density and coverage, resolving genomic composition ambiguities undetectable by traditional cytogenetic methods. The SSL markers enable real-time monitoring of chromosomal inheritance and recombination dynamics, addressing a fundamental challenge in distant hybridization-genomic instability. By facilitating early-generation screening, these markers significantly reduce dependence on labor-intensive phenotypic evaluations, accelerating the development of stable addition lines. For instance, the identification of *I. indigotica* chromosomal fragments in ‘Songyou No. 1’ demonstrates how marker-assisted selection optimizes trait combinations (e.g., coupling *B. napus* high yield with *I. indigotica* disease resistance or medicinal compound biosynthesis) while minimizing breeding cycles. This efficiency is further exemplified by the AMAC method, which employs SSL markers for rapid, high-accuracy detection of exogenous fragments, establishing a scalable framework for hybrid derivative analysis.

*I. indigotica*, commonly known as woad, is a traditional dye and medicinal plant in China. Its roots (i.e., Banlangen) and leaves (i.e., Daqingye) exhibit strong antiviral activity, as well as antibacterial, anti-endotoxin, anticancer, immune-regulatory, and blood-activating properties [46]. The creation of the full set of *B. napus*- *I. indigotica* addition lines [4,10,11,12] has facilitated research into the genes carried by the alien chromosomes of *I. indigotica* in the *B. napus* background and the metabolic products determined by these genes. This provides new materials and insights for studying genome structure, medicinal functional components, and disease resistance mechanisms. Kang et al. used five *B. napus*- *I. indigotica* monosomic addition lines (Mb, Mc, Md, Me, and Mf) and one disomic addition line (Dd) with sterile cytoplasm, cultured and propagated through tissue culture to prepare aqueous extracts from the above-ground parts. Metabolomics analysis and in vitro antiviral activity evaluations were conducted. Principal component analysis revealed that the disomic addition line Dd exhibited the greatest metabolic difference from the *B. napus* metabolome and demonstrated some inhibitory effects on the H1N1 influenza virus [10]. Further studies on the in vitro antiviral activity of *B. napus*- *I. indigotica* disomic addition lines with normal *B. napus* cytoplasm and seed-reproductive ability found that three materials (Dd, Df, and Dg) exhibited resistance to the highly pathogenic avian influenza virus H5N6, comparable to the widely used antiviral drug Oseltamivir, and even stronger than that of *I. indigotica* itself [9]. These findings suggest that alien chromosomes of *I. indigotica* carry genes for synthesizing specific medicinally active components, which can be expressed under the *B. napus* background. In the present study, we identified 32 genes located on the *I. indigotica* chromosomes I5 (c), I6 (g), and I7 (a) (Table S1), which are responsible for generating bioactive substances. Among these, 26 genes are located on chromosome I6 (g), and the antiviral strain ‘Songyou No. 1’ was derived from the hybridization of *B. napus*- *I. indigotica* monosomic addition line Mg with *B. napus* variety ‘Huashuang No. 3’ [10,12]. The discovery that chromosome I6 (g) of *I. indigotica* harbors a gene cluster associated with bioactive compound biosynthesis provides critical insights into the molecular basis of the antiviral activity observed in the hybrid line ‘Songyou No. 1’. This genomic configuration strongly suggests that the antiviral properties of ‘Songyou No. 1’—including efficacy against highly pathogenic H5N6 avian influenza virus—stem directly from the expression of I6 (g)-derived metabolites under the *B. napus* genetic background. These findings establish ‘Songyou No. 1’ as a promising multifunctional crop with applications spanning (1) Human nutrition (vegetable oil production), (2) Sustainable agriculture (antiviral phytochemicals enriched animal feed), and (3) pharmaceutical industries (large-scale production of bioactive precursors).

## 4. Materials and Methods

### 4.1. Materials and DNA Extraction

12 *I. indigotica* genotypes were collected across multiple provinces and cities in China, and they were used as positive controls for the genome specificity of the developed markers. 12 *Brassica* genotypes (*B. rapa*, *B. oleracea*, *B. juncea*, and *B. napus)* were used as a negative control, provided by the Industrial Crops Institute, Yunnan Academy of Agricultural Sciences. The genus hybrid ‘Songyou No. 1’ was used as the testing genotype, contributed by Huazhong Agricultural University Wuhan, China, which was the offspring of an intergeneric cross between *B. napus* and *I. indigotica* [11] (Table S7). The DNA extraction was performed using the CTAB method [47].

### 4.2. Genome Sequence Resource

The genome data for *I. indigotica* (Woad-v1.0) were obtained from the NCBI website (https://www.ncbi.nlm.nih.gov/Taxonomy/Browser/wwwtax.cgi?lvl=0&id=161756, accessed on 1 February 2020) [4] for whole-genome IP and SSR markers development. Additional reference genomes for *I. indigotica* and *Brassica* used in IP and SSR molecular markers electron PCR (ePCR) analysis included: the genome ASM1459570v1 downloaded from https://www.ncbi.nlm.nih.gov/assembly/GCA_014595705.1 (accessed on 15 September 2020), the genome ASM90040638v1, downloaded from https://www.ncbi.nlm.nih.gov/assembly/GCA_900406385.1 (accessed on 15 June 2019), the genome sequences of *B. rapa* (Brara_Chiifu_V3.0) [48], *B. oleracea* (Braol_JZS_V1.1) [49], and *B. juncea* (Braju_tum_V1.5) [50], downloaded from http://brassicadb.cn (accessed on 7 July 2022), the pan-genome sequences of *B. napus* was downloaded from the website http://cbi.hzau.edu.cn/bnapus (accessed on 10 September 2020) [20].

### 4.3. Development and Identification of I. indigotica Whole Genome Specific-Single-Locus IP and SSR Markers Based on the Method of AMAC

#### 4.3.1. Development and Analysis of *I. indigotica* Whole Genome IP and SSR Markers

Based on the AMAC methodology established in our previous study [24], whole genome IP and SSR markers were developed using the IPv2.0 tool [24] and MISA tool [51] with *I. indigotica* genome (Woad-v1.0).

IPv2.0 tool was employed to develop IP markers, which were independently developed by the Industrial Crops Research Institute, Yunnan Academy of Agricultural Sciences, registered under copyright number 2021SR0437322 in China. Executing the IPv2.0 tool required three inputs: (1) the reference genome (FASTA format), (2) the annotation file of the reference genome (GFF/GFF3/GTF format), and (3) a key parameter, an integer, specifying the maximum intron length threshold for IP markers development. The three inputs were the file of the ‘Woad-v1.0’ reference genome sequence, file of its corresponding annotation, and ‘500’, respectively, in this study. The IP_V2.0 pipeline automatically integrates Primer3.0 for primer design and ePCR tool for in silico amplification prediction through its Perl/Shell scripting framework.

The Microsatellite Identification Tool (MISA) was employed to identify SSR regions with the following parameter settings: unit sizes and minimum repeat thresholds set as 1-12, 2-6, 3-5, 4-5, 5-5, and 6-5, with interruptions (maximum difference between two SSRs) set to 100.

Primer3.0 was used for designing primers with the following parameter settings: the primer length was controlled between 18 and 24 bp, with an optimal size of 22 bp; the melting temperature was 58 °C to 65 °C, with an optimal temperature of 60 °C; the GC content was in the range of 40% to 60%; and the predicted PCR product length was in the range of 80–900 bp.

The ePCR tools were used to further refine primer selection and optimize the utility of the IP and SSR primers with the following parameter settings: 3 base pair mismatch, 1 base pair gap, 60 base pair margin, and a product size range of 80–1500 base pairs.

All other parameters were set as their default values.

#### 4.3.2. Prediction of Whole-Genome Single-Locus (SL) and Specific Single-Locus (SSL) Markers in *I. indigotica*

Based on in silico results from whole-genome IP and SSR primers, the IP and SSR markers predicted to amplify a single locus across all three *I. indigotica* reference genomes were categorized as *I. indigotica* genome SL-IP and SL-SSR markers, respectively. These markers were then subjected to ePCR analysis in the genomes of *Brassica* crops—including *B. rapa* (AA genome), *B. oleracea* (CC genome), *B. juncea* (AABB genome) and the pan-genome of *B. napus* (AACC genome). The parameters were identical to Section 4.3.1. The ePCR results were incorporated into a reverse selection process, wherein any *I. indigotica* SL-IP and SL-SSR markers expected to amplify in any of the *Brassica* reference genomes were excluded. Ultimately, only those markers that consistently amplified a single locus in all three *I. indigotica* genomes and also produced null ePCR products in all analyzed *Brassica* genomes were retained. These were predicted to be *I. indigotica* genome SSL-IP and SSL-SSR markers (in comparison to *Brassica* crops). The SSL markers located on chromosomes I1-I7 were selected for further experimental verification.

#### 4.3.3. Validation of *I. indigotica* Genome SSL-IP and SSL-SSR Markers

To validate the specificity of the predicted SSL-IP and SSL-SSR Markers, 15–20 primer pairs were randomly selected per chromosome according to its size and location information from the results of ePCR analysis. A total of 205 IP primers and 50 SSR primers were synthesized, and subsequently experimentally PCR amplification using DNA from 12 *I. indigotica* inbred lines and 12 *Brassica* crops materials, including three *B. napus* hybrid, two conventional *B. napus* types, two *B. juncea* types, two *B. oleracea* types, two *B. rapa* types, and one *B. napus* addition line (‘Songyou No. 1’) (Table S7), with four samples randomly selected from each group (I1-I4 and B1-B4). Markers demonstrating consistent amplification in all four *I. indigotica* materials but showing non-amplification in any *Brassica* samples were selected. These candidate markers were then PCR-amplified across all 12 *I. indigotica* samples (I1-I12) and 12 *Brassica* crop populations (B1-B12) to confirm their specificity as *I. indigotica* genome SSL markers.

The PCR reaction system was 20 µL:2.5 µL (50 ng/µL) of template DNA, 0.5 µL (10 µmol/L) of each forward and reverse primer, and 16.5 µL of T3 Super PCR Mix (Qingke Biotechnology Co., Ltd., Beijing, China). The PCR amplification program was: 98 °C for 2 min for pre-denaturation, then 98 °C for 10 s for denaturation, 58 °C for 12 s for annealing, and 72 °C for 12 s for extension, for a total of 35 cycles, followed by a final extension at 72 °C for 2 min, and storage at 4 °C. PCR products were separated using 1% agarose gel electrophoresis at a constant voltage of 160 V for 20 min. After electrophoresis, images were captured and saved using a gel imaging system (UVI Platinum/Explorer).

### 4.4. Detecting Exogenous Chromosomal Segment in ‘Songyou No. 1’

The ‘Songyou No. 1’ was used as the twelfth sample of *the Brassica* samples group (B12, Table S7) for detecting exogenous chromosomal segments from the *I. indigotica* genome. The interval of exogenous *I. indigotica* chromosomal segments was determined by SSL markers amplifying in ‘Songyou No. 1’ (see Section 4.3.3 for details).

### 4.5. Analysis of Genes Involved in Biosynthetic Pathways of Active Compounds Within Alien Chromosomal Segments from I. indigotica Genome in ‘Songyou No. 1’ and Development of Functional Markers

According to genes annotation of the reference genome of *I. indigotica*, 59, 66, and 32 genes involved in the biosynthetic pathways of terpenoids, lignans, and indole alkaloids were respectively obtained [4]. Further analysis was performed to estimate whether these genes were present in Songyou No. 1 depending on the location of these genes in the reference genome, Woad-v1.0 [4].

PCR and electrophoresis were employed to verify those SSL-IP markers within genes involved in synthetic pathways of three bioactive compounds. The verified SSL-IP markers were categorized as functional markers of corresponding genes.

### 4.6. Drawing Method

The circle diagram was created using OmicStudio tools with the default parameters (https://www.omicstudio.cn/tool, accessed on 6 February 2023). The genetic marker map was constructed using the online tool MG2C_V2.1 (http://mg2c.iask.in/mg2c_v2.1/index.html, accessed on 3 March 2022) [52], with default settings. Marker positions were determined according to the results of ePCR analysis based on the Woad-v1.0 reference genome sequence.

The Venn diagram was generated using the online interactive tool VENNY2.1 (https://bioinfogp.cnb.csic.es/tools/venny/index.html, accessed on 1 January 2015), was used to draw Venn’s diagrams.

## 5. Conclusions

This study successfully developed and partially validated a substantial set of *I. indigotica* genome SSL-IP and SSL-SSR markers. Through the AMAC methodology, we characterized one complete I6 chromosome and three chromosomal fragments (I1:1.17 Mb, I5:2.61 Mb, I7:1.11 Mb) derived from *I. indigotica* in ‘Songyou No. 1’. Additionally, we identified 32 biosynthetic pathway genes for active compounds located on chromosomes I1 (f), I3 (b), I5 (c), and I6 (g) of *I. indigotica*, subsequently developing seven functional markers in ‘Songyou No. 1’. These validated SSL markers and functional markers establish a technical foundation for implementing marker-assisted breeding strategies in medicinal plants via distant hybridization between I. indigotica and Brassica species.

## Figures and Tables

**Table 1:** The e-PCR results for IP and SSR marker loci in three genomes of *I. indigotica*.

Marker Type	Ref. Genome	e-PCR for 1 Locus	e-PCR for 2 Loci	e-PCR for 3 Loci	e-PCR for More Than 3 Loci	Total
IP	Woad-v1.0	90,636	18,513	5982	5171	120,302
	ASM1459570v1	89,686	14,837	3556	3357	111,436
	ASM90040638v1	58,785	12,364	2359	1229	74,737
SSR	Woad-v1.0	48,142	9237	3029	12,356	72,764
	ASM1459570v1	42,951	5905	1748	10,700	61,304
	ASM90040638v1	19,595	4999	1326	3541	29,461

**Table 2:** The e-PCR analysis for 41,003 pairs of SL IP and 11,742 SL SSR *I. indigotica* markers against different *Brassica* genomes.

Marker Type	Ref. Genome	e-PCR for 1 Locus	e-PCR for 2 Loci	E-PCR for 3 Loci	e-PCR for More Than 3 Loci	Total
IP	*B. napus* pagenome	3387	4999	3403	6444	18,233
	*B. rapa*	11,084	3153	607	129	14,973
	*B. oleracea*	9728	2502	406	108	12,744
	*B. juncea*	6203	6311	3025	3425	18,964
SSR	*B. napus* pagenome	375	392	235	302	1304
	*B. rapa*	785	175	30	5	995
	*B. oleracea*	674	136	17	3	830
	*B. juncea*	617	424	170	165	1376

**Table 3:** The distribution of 193 PCR-verified genome SSL-IP and SSL-SSR markers for the *I. indigotica* genome.

Chromosome	Total Length (Mb)	Counts of SSL Markers	Density of SSL Markers/Mb
I1	37.14	29	0.78
I2	36.13	26	0.71
I3	37.85	25	0.66
I4	34.44	26	0.75
I5	33.15	31	0.94
I6	38.22	26	0.68
I7	33.37	30	0.90

**Table 4:** Genes involved in the biosynthetic pathways of bioactive compounds from the alien fragments of *I. indigotica* in ‘Songyou No. 1’.

Metabolic Pathway	Gene ID	Chromosome	Start Position	End Position	Abbreviation	Description
	Iin18475	I5	10,901,212	10,904,010	SMO2	plant 4alpha-monomethylsterol monooxygenase
Terpenoids and sterols biosynthetic pathways	Iin21466	I6	23,170,011	23,173,893	DXS	1-deoxy-D-xylulose-5-phosphate synthase
	Iin23455		35,110,815	35,113,835	HDR	4-hydroxy-3-methylbut-2-en-1-yl diphosphate reductase
	Iin23689		36,492,488	36,493,670	GGPS	geranylgeranyl diphosphate synthase
	Iin23973		38,150,139	38,151,871		
	Iin23487		35,259,009	35,262,998	FDFT	farnesyl-diphosphate farnesyltransferase
	Iin23488		35,263,839	35,267,058		
	Iin23772		37,054,747	37,057,792	SQLE	squalene monooxygenase
	Iin22242		28,230,234	28,232,417	SMO1	plant 4,4-dimethylsterol C-4alpha-methyl-monooxygenase
Lignans and flavonoids biosynthetic pathways	Iin18350	I5	10,107,465	10,114,592	C4H	cinnamate 4-hydroxylase
	Iin18353		10,117,910	10,120,142		
	Iin18354		10,129,174	10,131,535		
	Iin18531		11,574,246	11,575,487	DIR	Dirigent protein
	Iin22645	I6	30,755,729	30,757,995	CCoAOMT	caffeoyl-CoA O-methyltransferase
	Iin23419		34,931,455	34,933,174		caffeoyl-CoA O-methyltransferase
	Iin23442		35,032,027	35,034,075	CAD	cinnamyl-alcohol dehydrogenase
	Iin23794		37,202,962	37,205,920		
	Iin23795		37,207,004	37,209,625		
	Iin23796		37,216,859	37,219,436		
	Iin23889		37,744,686	37,746,684		
	Iin20509		5,966,754	5,967,454	DIR	Dirigent protein
	Iin21107		19,140,553	19,141,528		
	Iin22345		29,074,569	29,075,138		
	Iin22360		29,163,765	29,164,388		
	Iin21123		19,340,771	19,343,941	PLR	pinoresinol/lariciresinol reductase
	Iin24104	I7	442,462	444,628	CCR	cinnamoyl-CoA reductase
Indole alkaloids biosynthetic pathways	Iin22896	I6	31,982,635	31,989,149	DDC	aromatic-L-amino-acid/L-tryptophan decarboxylase
	Iin22479		29,865,590	29,868,588	TAA	L-tryptophan---pyruvate aminotransferase
	Iin21070		18,226,026	18,228,437	YUCCA	indole-3-pyruvate monooxygenase
	Iin22904		32,050,342	32,052,237		
	Iin23276		34,170,537	34,172,463		
	Iin23829		37,348,774	37,350,974	CYP79B	tryptophan N-monooxygenase

**Table 5:** Functional markers of genes related to bioactivity compounds.

Functional Marker	Gene ID	Forward Primer Sequence (5′-3′)	Reverse Primer Sequence (5′-3′)
I6. YUCCA-1	Iin21070	GACCGGTTCTTGTTACGCAT	CATCAACAAACTCTGCCGAA
I6. TAA	Iin22479	CACTTTAACCATGCAAACGC	TGAACGTTGCAATTTCCTTG
I6. CAD-1	Iin23442	ACGAGACAACCAACTCCGAC	TCGGCATGTCTAATTACCCC
I6. CAD-4	Iin23796	CAAAAGTTCAGAAGGAGGCG	ACATTCCCCAATCGTTCTTG
I6. CAD-5	Iin23889	TCTCCTTCATCCCTCCAATG	GCAAAGAAGACGAAGCCATC
I6. GGPS-2	Iin23973	ACAAGATCAGGAGGGGTGTG	GGAGATCAACCAGAAGCTCG
I7. CCR	Iin24104	CTTGGAGAGAGGCTACACCG	CTTGACCTTTGCTTTAGCGG

## Data Availability

All data were shown in Tables and Figures in the main text or Supplemental Files.

## Supplementary Materials

The following supporting information can be downloaded at: https://www.mdpi.com/article/10.3390/ijms26052091/s1.
